# Unraveling the Converging Roles of ASC-Dependent Inflammasomes, Interleukin-1 Superfamily Members, Serum Amyloid A, and Non-Sterile Inflammation in Disease Pathology and Fibrosis in Inflammatory Bowel Disease and Primary Sclerosing Cholangitis

**DOI:** 10.3390/ijms26168042

**Published:** 2025-08-20

**Authors:** Marco Losa, Marlene Schwarzfischer, Marc Emmenegger, Marianne R. Spalinger, Gerhard Rogler, Michael Scharl

**Affiliations:** 1 Department of Gastroenterology and Hepatology, University Hospital Zurich, 8091 Zurich, Switzerland; marianne.spalinger@usz.ch (M.R.S.); gerhard.rogler@usz.ch (G.R.); 2 Department of Gastroenterology and Hepatology, University Hospital Zurich, University of Zürich, 8006 Zurich, Switzerland; marlene.schwarzfischer@usz.ch; 3 Division of Medical Immunology, University Hospital Basel, 4031 Basel, Switzerland; marc.emmenegger@usb.ch

**Keywords:** inflammatory bowel disease (IBD), primary sclerosing cholangitis (PSC), chronic inflammation, fibrosis, innate immunity, inflammasomes, apoptosis-associated speck-like protein containing a caspase recruitment domain (ASC), inflammasome adapter, pyrin domain (PYD), caspase recruitment domain (CARD), Interleukin (IL)-1 superfamily, IL-1, IL-1 receptor(R)1, IL-18, IL-33, caspase-1, serum amyloid A (SAA), intestinal microbes, gut microbiome, non-sterile inflammation, therapeutic agents

## Abstract

Inflammatory bowel disease (IBD) and primary sclerosing cholangitis (PSC) are chronic immune-mediated inflammatory diseases (IMIDs) that affect the gastrointestinal and hepatobiliary systems. They are characterized by persistent inflammation, potentially progressive fibrosis, and an elevated risk of developing cholangiocarcinoma and colorectal cancer. IBD and PSC share phenotypical, genetic, and immunological features, largely due to the central role of immune cell dysregulation. Despite their increasing global prevalence, the underlying drivers remain poorly understood, and effective treatment options are still lacking. Efforts towards an improved comprehension of their pathogenic mechanisms are therefore pivotal. Emerging evidence highlights the role of canonical ASC-dependent inflammasomes—multiprotein bioactive Interleukin (IL)-1-producing complexes of the innate immune system—and serum amyloid A (SAA) as key structures of gastrointestinal and hepatobiliary inflammation, tissue remodeling, stromal crosstalk, and fibrosis. In this review, we explore immunological connections and analogies between IBD and PSC, highlighting the converging roles of canonical ASC-dependent inflammasomes, the IL-1 superfamily, SAA, and sustained gut microbiota-driven chronic inflammation in disease pathology and their surging potential as therapeutic targets across the gut–liver axis.

## 1. Introduction

Primary sclerosing cholangitis (PSC) is a progressive liver disease characterized by chronic inflammation and fibrosis of the bile ducts, leading to cholestasis, cirrhosis, and premature death [1]. Globally, PSC is a rare disease with a prevalence of approximately 14 per 100,000 individuals, with higher rates reported in Northern Europe and North America [2]. A strong association with inflammatory bowel disease (IBD), particularly ulcerative colitis (UC), is observed in 60–80% of PSC patients, and is termed PSC-associated IBD (PSC-IBD), strongly suggesting shared underlying pathogenic mechanisms [3,4,5,6]. IBD, including UC and Crohn’s disease (CD) affects over 6.8 million people worldwide, corresponding to a prevalence of 84 per 100,000 individuals with an unexplained increasing global burden. IBD and PSC are significantly more common in industrialized countries [7,8].

Whereas IBD symptoms include bloody diarrhea, abdominal cramping, tenesmus, and malnutrition, PSC commonly presents with fatigue, pruritus, and jaundice. A typical laboratory finding includes elevated fecal calprotectin levels in IBD and elevated cholestasis parameters in PSC [9,10]. Moreover, PSC is usually accompanied by mildly elevated transaminases in the absence of elevated humoral markers such as total IgG or IgM concentrations—unlike in primary biliary cholangitis (PBC) [11].

Collectively, both diseases are characterized by an uncontrolled chronic inflammatory state, tenfold increased risk of hepatobiliary and intestinal malignancies (cholangiocarcinoma and colorectal cancer), stromal and immune cell activation, excessive extracellular matrix (ECM) deposition, termed fibrosis, and progressive narrowing of affected intestinal segments (greater stricture tendency in PSC-IBD) or bile ducts, respectively [12,13,14,15,16,17,18,19,20,21]. Although intra-and extrahepatic bile duct fibrosis is the hallmark of PSC, only about 20% of IBD patients develop disproportionate intestinal ECM synthesis and fibrotic complications, including tenacious ulceration or fistulae, which often necessitate surgical resection [22,23,24]. Notably, patients with PSC-IBD exhibit some distinct clinical and histological characteristics (compared to PSC or IBD alone), including reduced response to conventional IBD therapies and rectal sparing [25,26].

Overall, intestinal and hepatobiliary fibrosis is caused by activated stromal cells such as (myo)fibroblasts. However, the precise molecular mechanisms and signaling cascades underlying hepatobiliary and intestinal stromal remodeling and fibrosis still remain elusive [27].

In recent years, increasing attention has focused on the role of the innate immune system—especially myeloid cells—in the pathogenesis of PSC and IBD. Canonical apoptosis-associated speck-like protein containing a caspase recruitment domain (ASC)-dependent inflammasomes and their signaling cascade have emerged as key regulators of inflammatory responses through activation and production of highly pro-inflammatory cytokines, including Interleukin (IL)-1 and IL-18, which contribute to chronic inflammation in both bile ducts and intestinal mucosa [28,29,30]. Their roles and exploration in PSC and IBD pathogenesis as pivotal mediators of inflammation and fibrosis have therefore gained attention [3,31,32,33,34]. In essence, these findings indicate that impaired regulation or hyperactivation of innate immune pathways—particularly inflammasomes—underlie PSC and IBD pathology. If so, targeting theses signaling cascades could offer novel therapeutic opportunities.

This review outlines the structural and functional aspects as well as the key knowledge gap of IL-1-producing ASC-inflammasomes—specifically the convergence of ASC-inflammasome pathways and serum amyloid A (SAA)—and gut microbiota in the pathogenesis of PSC and IBD. We discuss recent pre- and clinical therapeutic approaches and how these inflammasome pathway elements interplay and contribute to sustained, microbiota-driven inflammation and fibrosis across the gut–liver axis.

Ultimately, elucidating the molecular signatures of inflammasome signaling-mediated inflammatory cascades in PSC and IBD may pave the way for novel therapeutic strategies to remedy the burden imposed by these irreversible and chronic inflammatory diseases affecting the gastrointestinal as well as the hepatobiliary system.

## 2. The Acute-Phase Response Mediates Tissue Restoration upon Injury

In healthy individuals, injuries to the intestinal mucosa or liver trigger an organized wound-healing process mediated by an acute-phase response (APR) within 24 h upon insult. An APR is initiated by tissue injury, pathogen invasion (e.g., owing to transitory viral infections), or an (auto)inflammatory state that triggers an innate immune system-mediated response. This results in the hepatocellular synthesis and secretion of acute-phase proteins such as SAA, C-reactive protein (CRP), ferritin, and haptoglobin, which support tissue repair and integrity restoration [35,36,37]. These proteins are transcriptionally regulated by pro-inflammatory cytokines—originating from the site of the insult—such as tumor necrosis factor alpha (TNF-α), IL-6, and IL-1β released into the circulation by immune and capable tissue cells at the injury site. Innate immune responses are not pathogen-specific but contingent on various tissue properties like tissue barriers (e.g., epithelial tight junctions, mucus-secreting cells, mucosal layers), a specified set of proteins (complement factors and defensins), and leukocytes such as granulocytes, macrophages, mast cells, and lymphocytes. Innate immune cells detect conserved microbial and danger-associated patterns and molecules via pattern recognition receptors (PRRs) that elicit inflammasome activation and an accurate APR [38]. Canonical inflammasomes are large, modular multiprotein complexes found in most cells of the body but highly expressed in myeloid immune cells. They assemble rapidly and reliably to convert danger signals into a caspase-1-dependent pro-inflammatory cytokine response in which caspase-1 mediates the proteolytic maturation of IL-1 superfamily members IL-1β and IL-18 as well as the pore-forming protein gasdermin-D, which mediates the release of these cytokines into the extracellular space and the circulation (see first figure in [39,40]), promoting adaptive immune activation and tissue repair through immune–stromal cell interactions and crosstalk [29,41,42,43].

## 3. The Central Inflammasome Adapter ASC Couples Pyrin (PYD) and Caspase Recruitment Domain (CARD)-Containing Inflammasome Sensors with Interleukin-1 Bioactivating Effector Machinery

Functional inflammasomes consist of a PRR that acts as a sensor of danger signals, an adapter (i.e., ASC), and an effector molecule (i.e., caspase-1). These three elements assemble into highly organized high-molecular-weight complexes in a tightly regulated manner upon sensor activation [44,45,46].

**Figure 1:**
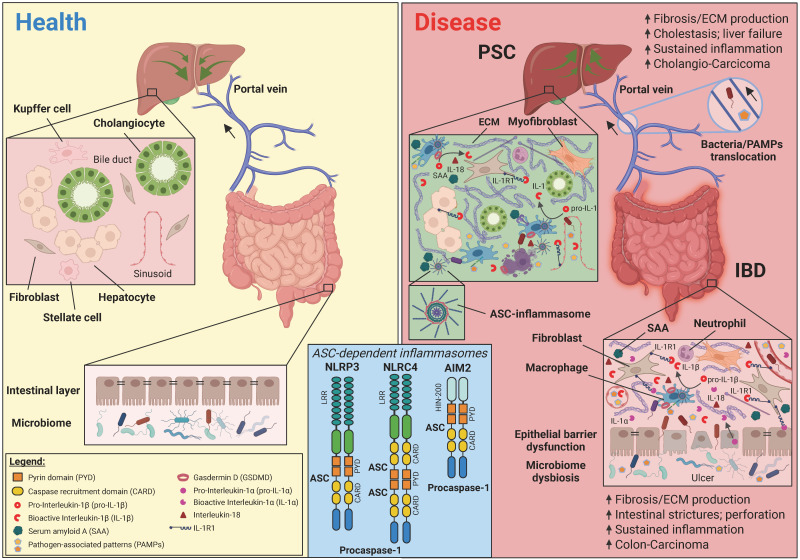
ASC-dependent inflammasomes in PSC and IBD pathology. Homeostasis and healthy liver and intestinal mucosal state (**left**) and inflamed/fibrotic disease state (**right**). A modular structure illustration of the best-known ASC-dependent inflammasomes (**middle box**).

PRRs are germline-encoded and membrane-bound—such as receptor kinases, Toll-like receptors (TLRs), and C-type lectin receptors—or cytoplasmic, including nucleotide-binding oligomerization domain (NOD)-like receptors (NLRs) and RIG-1 (retinoic acid-inducible gene 1)-like receptors. These receptors often contain conserved domains such as leucine-rich repeat (LRR), pyrin (PYD), and caspase activation and recruitment domains (CARD) that allow homophilic protein–protein interactions (PPI). External danger signals recognized by PRRs, known as pathogen-associated molecular patterns (PAMPs), are conserved molecular features found in microbes. These signals initiate non-sterile inflammatory responses, typically triggered by microbial presence. In contrast, sterile non-microbial-induced inflammation is caused by damage-associated molecular patterns (DAMPs) and lifestyle-associated molecular patterns (LAMPs) [38,47], which originate from host-derived or environmental stressors. Depending on the PRR involved, inflammasomes are categorized into the NLR and the non-NLR HIN200 (hematopoietic interferon-inducible nuclear antigens with 200 amino-acid repeats) family. NLRP1/3/6/7 (NOD, leucine-rich repeat (LRR), and PYD (domain)-containing) and NLRC4/5 (NOD, LRR, and CARD (domain)-containing-4/ICE protease-activating factor) belong to the former family, whereas absent in melanoma-2 (AIM2) belongs to the latter family. NLRP1/3, NLRC4/IPAF, and AIM2 inflammasomes are canonical inflammasomes that depend on the ASC adaptor protein to ultimately activate caspase-1 (Figure 1).

Danger signal-sensing proteins of the NLRP [48,49] and AIM (HIN200) [50] families contain a PYD, which enables homophilic PPI and self-association via the PYD domain of the central inflammasome adapter and heterodimer ASC [41,51,52,53]. In contrast, the NLRC family members possess a CARD (instead of a PYD), allowing them to recruit caspase-1 either directly or via the ASC adaptor (Figure 1). Recent studies have shown that NLRP3 inflammasome activation and ASC recruitment are initiated by danger signal-dependent NLRP3 palmitoylation and phase separation, triggered by decreased PRR solubility [54].

Historically, ASC was first described in 1999 as a self-assembling protein encoded by the *PYCARD/ASC* gene [55]. Full-length ASC comprises two death domains: an 89-amino-acid (aa) N-terminal PYD and an 83 aa C-terminal CARD, connected by a 23 aa hinge region [42,56]. A few years after its discovery, ASC was shown to function as a key adaptor for caspase-1-mediated IL-1β production and was found to be expressed in several immune cell types and a variety of stromal and epithelial cells including monocytes, macrophages, neutrophils, natural killer cells, T cells, and intestinal epithelial cells [44,56,57]. Moreover, lipopolysaccharide-injected *Pycard*-deficient animals demonstrated improved survival in comparison to wildtype littermates, likely due to the absence of a detrimental hyperinflammatory innate immune response [58,59].

Mechanistically, the adaptor protein ASC, upon PRR-mediated recruitment, undergoes a unified polymerization mechanism during the assembly of ASC-dependent inflammasomes and exhibits both extracellular (in form of ASC specks) and prion-like self-assembly properties that propagate inflammation [51,52,60]. Once ASC is recruited by PRRs, it interacts and oligomerizes with the CARD domain of the effector protein pro-caspase-1. This clustering brings multiple pro-caspase-1 molecules into close proximity, enabling their auto-catalytic cleavage into active capsase-1 [42]. Subsequently, effector caspase-1, an active hetero-tetramer consisting of two p10 and two p20 subunits derived from two cleaved pro-caspases-1, processes pro-IL-1β into its mature, bioactive form (IL-1β) [28,29,61,62,63]. In parallel, active caspase-1 cleaves gasdermin-D to generate an N-terminal fragment that forms membrane pores, facilitating the release of other IL-1 superfamily members such as IL-18 (together with IL-1β and IL-33) into the extracellular space and circulation, and inducing mediating pyroptotic cell death [41,64].

In summary, ASC functions as the central adaptor linking ASC inflammasome sensors to the effector molecule pro-caspase-1. In this process, the assembly of an inflammasome PRR, ASC, and pro-caspase-1 forms a multimeric high-molecular-weight protein complex—the inflammasome—which transduces and amplifies danger signals of various origins into a potent pro-inflammatory cytokine response. Beyond its role in microbial defense, ASC has been linked to tumor suppression, cerebrovascular injury response, and inflammatory diseases including PSC and IBD [38,44,46,52,65,66,67,68].

## 4. Dual Functions of IL-1 Family Members in Balancing Inflammation and Repair Contribute to Epithelial and Mucosal Homeostasis

IL-1, comprising two main members of the IL-1 superfamily—IL-1α and IL-1β—is a major contributor to the inflammatory response, tissue regeneration, and maintenance of homeostasis following epithelial or endothelial injury triggered by DAMPs and PAMPs across various tissues [29,41,51,69]. Although the precise molecular mechanisms remain unclear, sustained ASC-inflammasome activation and persistent inflammation can induce fibrotic processes in various organs [70,71,72,73,74].

Mechanistically, IL-1 cytokines signal exclusively through IL-1 receptor 1 (IL-1R1), which is expressed by a variety of cell types, including endothelial cells and fibroblasts [75,76]. Fibrosis of the bile ducts and intestinal wall may therefore be driven—though not exclusively—by IL-1R1 signaling, stromal cell activation, and excessive ECM synthesis and deposition [63,75,77,78,79,80,81]. Moreover, disruptions of the intestinal barrier, as seen in IBD-related ulcerations, are associated with increased fibrosis (e.g., in Crohn’s disease). It appears that hyperinflammatory immune cell states—sustained by non-sterile inflammation—elevate tissue IL-1 levels, which may disrupt stromal crosstalk and promote IL-1R1-mediated myofibroblast activation, dysfunctional wound healing, and pro-fibrotic processes [76,81,82,83,84]. Supporting the pathological role of IL-1 in fibrosis, studies have shown that the absence of IL-1α and IL-1β inhibits liver fibrosis in steatohepatitis. Additionally, evidence from ischemic cardiac injury models implicates IL-1 signaling in fibroblasts as a driver of inflammation-induced tissue remodeling and fibrosis [85,86].

Beyond their inflammatory roles, IL-1 family members also play crucial roles in mucosal and epithelial regeneration and homeostasis by regulating immune cells in gastrointestinal disorders [87]. One of the first studies to demonstrate the dual role of IL-1 in colitis was conducted by Cominelli and colleagues [88]. They showed that a low dose of IL-1β administered 24 h before dextran sulfate sodium (DSS) exposure protected against colitis, whereas administration just 30 min prior to DSS initiation did not confer protection. Furthermore, IL-18 has been shown to be essential for mucosal homeostasis: mice deficient in *Nlrp3*, *Pycard*/*Asc*, *caspase-1*, *IL-18*, or *IL-18R*—all components of the inflammasome pathway—are highly susceptible to DSS-induced colitis. Loss of these components leads to compromised epithelial integrity, bacterial translocation, enhanced leukocyte infiltration, and increased chemokine production—effects that can be ameliorated by exogenous IL-18 administration [89,90]. Since IL-18- and IL-18R-deficient mice are more susceptible to DSS than their wildtype littermates, IL-18—released from intestinal epithelial cells—appears to protect against colitis by promoting tissue repair and maintaining intestinal lining homeostasis [90].

These dual cytokine functions underscore the importance of tightly regulated, temporally dynamic cytokine activity during wound healing. In this context, it has been shown that IL-1 neutralization during the acute phase of murine colitis exacerbates inflammation and delays recovery from DSS-induced injury. In contrast, IL-1 blockade in the chronic phase of colitis has no effect [91]. This suggests that IL-1 has reparative functions during acute inflammation but exerts pro-inflammatory and pro-fibrotic effects during chronic disease. To our knowledge, no study has yet addressed the dual roles of IL-1 family cytokines in PSC pathology. 

In summary, IL-1 family members—particularly those from ASC-dependent inflammasome pathways—modulate the balance between inflammation and repair depending on disease context and cellular state in IBD.

## 5. ASC-Dependent Inflammasomes and Interleukin-1 Exert Analogous Roles in Inflammation and Fibrosis in PSC and IBD Pathology

Fibrotic disease states—including hepatobiliary and intestinal compartments in PSC and IBD—share common features of aberrant innate immune response and IL-1R1 signaling.

In PSC, multiple cell types—including cholangiocytes, hepatic stellate cells (HSCs), Kupffer cells, and immune cells such as T cells and macrophages—contribute to disease progression [1]. Monocytes from PSC patients exhibit hyperresponsive innate immune states, producing elevated IL-1β levels upon microbial stimulation, indicating inflammasome dysregulation in PSC pathology [92]. Numerous inflammation-associated mucosal cell populations—immune, stromal, and epithelial compartments—have also been identified in the IBD intestine, supporting their roles in disease pathogenesis and potential as biomarkers [76,93,94,95]. Myeloid cells, particularly macrophages, are strongly implicated in both PSC and IBD [96,97,98]. They are key expressers of inflammasome components and have been described to promote ECM synthesis by driving myofibroblast activation, migration, and proliferation, especially in IBD [94,96,99].

A study by Miyamoto and colleagues demonstrated that ablation of macrophages—specifically those expressing a scavenger receptor involved in sequestering DAMPs and PAMPs—led to uncontrolled inflammation and PSC-like phenotypes, highlighting the significance of innate immune cells in disease mechanisms [100]. Similar to lamina propria macrophages in IBD, Kupffer cells—the liver’s resident macrophages—are activated by microbial antigens and bile acids. Upon stimulation they release cytokines like TNF-α and IL-1β, which amplify inflammation and stimulate hepatic stellate cells to differentiate into fibrillogenic ECM-producing myofibroblasts via TGF-β and inflammasome-derived signals. This may eventually result in intrahepatic and extrahepatic bile duct fibrosis, while in IBD, similar mechanisms result in intestinal wall fibrosis (Figure 1).

Cholangiocytes—epithelial cells linking bile ducts—also play a central role in PSC [101]. Equipped with PRRs such as TLRs and NLRs, cholangiocytes can sense PAMPs and DAMPs. Upon activation, they secrete cytokines and chemokines such as IL-6 and CCL2, facilitating immune cell recruitment and sustaining inflammation. Chronic inflammatory signaling can also trigger epithelial-to-mesenchymal transition in cholangiocytes, contributing to fibrotic tissue remodeling. Immune cell infiltration, particularly by CD4+ and CD8+ T cells, exacerbates bile duct inflammation, while dysfunctional regulatory T cells (Tregs) fail to maintain immune homeostasis. Neutrophils, recruited by chemokines such as CXCL8, inflict further bile duct injury through the release of reactive oxygen species and neutrophil extracellular traps.

As abovementioned, epithelial barrier malfunction and a complex interplay of genetic, gut microbial composition, and sensing, as well as immunological factors, influence inflammasome activity and IBD severity and fibrosis [24,102]. Genome-wide association studies (GWAS) have associated polymorphisms in inflammasome-related genes—such as NLRP3, CARD9, and NOD2 [17,103]—with increased IBD risk, particularly in Crohn’s disease. Notably, NOD2 mutations correlate with a higher incidence of colorectal cancer in IBD patients. While inflammasome signaling is essential for maintaining intestinal homeostasis, its dysregulation—often due to genetic mutations or polymorphisms—promotes hyperinflammation and fibrosis. Activated fibroblasts and mesenchymal stem cells contribute to extracellular matrix remodeling and chronic intestinal injury. Moreover, IL-1R2—a decoy receptor for IL-1—has been identified as a susceptibility locus for IBD, further emphasizing the pathogenic relevance of IL-1 signaling as it ceases IL-1-driven processes [104,105,106]. Importantly, shared polymorphisms in IBD and PSC have been identified [107,108]. Among others, genetic risk loci related to host–microbiota interaction and immune dysregulation reinforce the role of inflammasomes in PSC-IBD [109,110,111], reflecting the overlapping distinct pathogenic mechanisms described above.

In conclusion, ASC-dependent inflammasomes are key modulators of innate immune responses in PSC and IBD. Their activation links microbial sensing with downstream inflammatory and fibrotic processes and stromal crosstalk, impacting a diverse array of cell types including cholangiocytes, hepatic stellate cells, macrophages, (myo)fibroblasts, endothelial cells, and intestinal epithelial cells. IBD and PSC remain complex multifactorial diseases shaped by genetic predisposition, environmental factors, gut microbiota imbalances, and immune dysregulation. Disruptions in epithelial barrier function, immune tolerance, and microbial recognition perpetuate chronic intestinal and hepatobiliary inflammation and aberrant stromal crosstalk.

## 6. Serum Amyloid A Proteins Represent Damage-Associated Molecular Patterns and Perpetuate Chronic Inflammation

Upon an inflammatory stimulus—primarily DAMPs or PAMPs—acute-phase reactants such as SAA proteins are secreted into the circulation, mainly by hepatocytes, under the transcriptional regulation of diverse pro-inflammatory cytokines including IL-1, IL-6, and TNF [112]. To some extent, extrahepatic SAA synthesis can also occur in adipocytes, epithelial cells, and endothelial cells throughout most human organs [113,114,115]. Human SAA and its isoforms consist of 103–104 amino acids (aa) and share high sequence homology. They belong to the group of high-density lipoprotein (HDL)-associated apoproteins and play an important role in HDL metabolism and tissue remodeling in vertebrates [116,117,118]. The human genome contains four SAA genes (*SAA1*, *SAA2*, *SAA3*, *SAA4*), located on chromosome 11p15.1 [119]. The *SAA1* and *SAA2* genes are highly inducible in an APR, while *SAA3* is a pseudogene and *SAA4* seems to be constitutively expressed. Following induction, SAA serum concentrations can increase up to 1000-fold within 24 h of APR onset [37]. SAA and its isoforms mediate immune activation and link APR response to long-term tissue remodeling dynamics. SAA proteins display context-dependent functions, promoting immune defense and tissue regeneration under certain conditions. However, they can also exacerbate chronic inflammatory pathologies—including colitis-associated cancer—through crosstalk with PRRs and metabolic pathways [120,121]. According to Ye and colleagues [122,123], SAA activates a variety of innate immune receptors such as the PRRs TLR2/4, FPR2 (formyl peptide receptor 2), and the adenosine triphosphate (ATP) receptor P2X_7_, as well as the HDL receptor SR-BI (scavenger receptor B1). SAA is an amyloidogenic protein that contributes to inflammatory amyloidosis by forming pathogenic and extracellular deposited amyloid A (AA) fibril aggregates. These aggregates distort tissue architecture and cellular physiology, particularly in the liver, spleen, kidneys, and heart [124,125]. Peripheral SAA aggregation has further been shown to be promoted by an ASC-mediated innate immune mechanism, independent of IL-1 signaling [52]. Over recent decades, high SAA levels have been implicated in many chronic inflammatory conditions, such as PSC [126], IBD [115], and others [127,128,129]. Lee and colleagues showed that SAA1 and SAA2 secretion by intestinal epithelial cells promotes a pathogenic, pro-inflammatory T-helper (Th) 17 cell differentiation program [115] and serves as a prognostic marker in IBD [130,131]. Therefore, SAA plays a critical role in leucocyte function, migration, and proliferation, as well as the transformation of fibroblasts and endothelial cells, processes potentially leading to fibrosis and strictures [120,129,132,133,134].

## 7. Microbiota–Inflammasome Crosstalk Sustains Chronic Inflammation and Fibrosis by Pathogen-Associated Molecular Patterns

The gut–liver axis, of which the microbiome composition forms a crucial part, plays a vital role in PSC associated with IBD. One proposed mechanism for sustained inflammation involves an altered intestinal epithelial barrier and increased permeability, commonly referred to as “leaky gut” [135,136,137,138,139,140]. This condition permits microbiota and their associated antigens to surpass the intestinal epithelial barrier, the liver, and bile fluids via the portal vein, triggering and provoking pathogen responses and inflammasome activation of innate immune cells along the gut–liver axis [31,141]. Conversely, microbial dysbiosis (i.e., *Bacteroidetes*, *Firmicutes*, *Enterobacteriaceae*) may exacerbate inflammation, as certain bacterial strains isolated from PSC-IBD patients have been shown to disrupt the intestinal epithelial barrier by inducing pore formation [142,143,144,145]. Moreover, GWAS have identified susceptibility loci in genes regulating immune responses, such as HLA-B08, DRB103, MST1, and FUT2, supporting the central role of immune cell trafficking and microbial sensing in PSC development [146]. Dysbiosis of the gut microbiome perpetuates immune activation through microbial products (e.g., lipopolysaccharide and bacterial outer membrane vesicles) which engage TLR4 and NLRP3 on hepatic innate immune cells, linking intestinal mucosal alterations to hepatic pathology [31,147]. Furthermore, in addition to their key role in host defense against bacteria and fungi, Th17 cells are implicated in the pathogenesis of autoimmune and chronic inflammatory diseases entailing fibrosis. Microbe-stimulated monocytes have been shown to induce chemokine (CCL-2 and CCL-20) production by cholangiocytes that drive Th17 responses and inflammation by recruiting Th17 cells and inflammatory monocytes into portal tracts in PSC [92,148]. Moreover, altered bile acid metabolism—often due to microbiome changes—can modulate inflammation and therefore promote PSC cholestatic liver injury by altering bile acid receptor farnesoid X receptor (FXR) signaling [149,150]. Lastly, it has been reported that bile acids are able to dampen NLRP3 inflammasome activity by G protein-coupled bile acid receptor 1 (GPBAR1, also known as TGR5) interaction [151].

## 8. Emerging Therapeutic Strategies Targeting ASC-Inflammasomes, Interleukin-1 Family Members, Serum Amyloid A, and the Gut Microbiome in PSC and IBD

**Figure 2:**
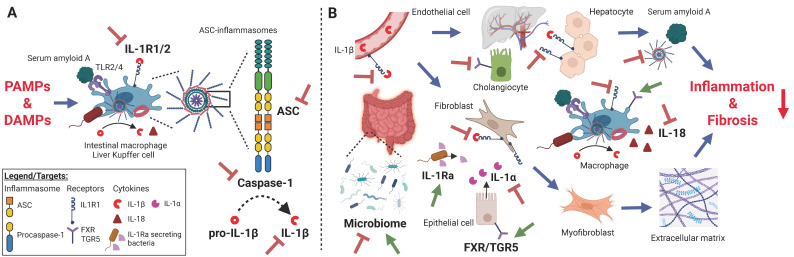
Promising targets along the ASC-inflammasomes and Interleukin-1/18 signaling cascade reported in recent pre- and clinical IBD and PSC treatment approaches. (**A**) Schematic of myeloid cell-derived ASC-inflammasomes and molecule blockade. (**B**) Representation of presumed inhibitory (inhibitory signs) and supplementary/agonism (green arrows) approaches among mucosal and hepatobiliary compartments along the gut–liver axis. Of note, agents may act in various cell types simultaneously. Details on described agents and their mechanism are listed in Table 1.

As described earlier, the pathogenesis of PSC and IBD and their relation remains poorly understood. However, there is growing evidence that a dysregulated innate immune system is heavily involved. Associated cytokines and cytokine-signaling mediators have been targets of successful IBD therapies, such as TNF, IL-12/IL-23, and janus-kinases (JAKs). However, a significant proportion of IBD patients will not respond or will lose response to available medications. A recent meta-analysis reported limited treatment efficacy of biologics targeting TNF, α4β7-Integrin, and JAK signaling—such as infliximab, adalimumab, vedolizumab, and tofacitinib—in PSC-IBD, underscoring the inherent difference from sole IBD and the incomplete understanding of the PSC-IBD disease entity. Conversely, this suggests that targeting ASC-dependent inflammasome pathways may offer a promising therapeutic avenue in the management of PSC-IBD [81,102,152,153,154]. Within the last decade, inflammasome-targeting agents, such as oridamin, tranilast, or dapansutrile (OLT1177), and various natural compounds have been described [155,156,157]. Profound exploration of therapeutic approaches targeting the ASC-inflammasome cascade, including immune and stromal cell types as well as the IL-1 superfamily cytokines and their receptors, has recently gained attention and yielded promising data in PSC and IBD research and treatment (Figure 2, Table 1).

### 8.1. ASC-Targeting Agents

Therapeutic strategies aimed at inhibiting inflammasome activation are of great interest in the context of PSC and IBD. A review about inflammasome-inhibiting agents—primarily those targeting NLRP3—shows clear beneficial effects in attenuating intestinal inflammation [155]. Agents such as MCC950, a rather selective NLRP3 inflammasome inhibitor [177,178], have shown promise in animal models of liver disease [179], reducing hepatic inflammation and fibrosis [180]. While these findings are encouraging, clinical trials are necessary to assess the safety and efficacy of MCC950 administration in patients with PSC. In murine models of IBD, MCC950 significantly reduced spontaneous colitis by inhibition of ASC oligomerization [158,159]. More recent agents have also gained attention through promising preclinical studies involving IBD models. One of these agents is decursinol angelate (DA), a natural compound that was found to inhibit the activation of the NLRP3 inflammasome—without caspase-1 activation—leading to reduced pyroptosis and inflammation in the colon in DSS-induced colitis in mice [160]. A recent study showed that atranorin, a natural compound, binds directly to ASC, inhibiting its oligomerization and subsequent NLRP3 inflammasome activation. Atranorin administration protected against NLRP3 inflammasome-driven diseases and ameliorated colitis in vivo by improving epithelial barrier function and reducing IL-1β and IL-18 production [161]. A dual-acting synthetic molecule and inhibitor termed 10 v has recently been suggested to attenuate in vivo induced-colitis by disrupting ASC interaction for proper NLRP3 and AIM-2 function as well as signal transducers and activators of transcription (STAT) signaling pathways [162]. Moreover, it has been shown that the small molecule ionidamine directly targets ASC, disrupting its interaction with pro-caspase-1 and preventing inflammasome assembly in neuroinflammatory models, suggesting its potential applicability in IBD and PSC [181], but further studies are needed to evaluate the effects of these agents.

### 8.2. Caspase-1 Inhibitors

Caspase-1 is the effector molecule elicited upon ASC-dependent inflammasome activation, leading to the maturation and release of pro-inflammatory cytokines such as IL-1β and IL-18. Caspase-1 knockout mice exhibited significant protection and against both acute and DSS-induced colitis—demonstrated by increased colon length, reduced weight loss, diarrhea, and rectal bleeding—as well as reduced IL-1β and IL-18 levels in total colon cultures of experimental mice compared to wild-type controls [182]. Inhibiting caspase-1 has therefore been proposed as a therapeutic strategy to mitigate inflammation in PSC with or without IBD. Belnacasan (VX-765) is an orally bioavailable prodrug that inhibits caspase-1 activity. Preclinical studies have demonstrated that VX-765 administration effectively reduces IL-1β secretion and attenuates inflammation in models of inflammatory diseases and alleviates DSS-induced colitis in mice by suppressing caspase-1 mediated pyroptosis [163,183,184]. However, its specific efficacy in PSC has yet to be thoroughly investigated.

### 8.3. Interleukin-1α- and Interleukin-1β-Blocking Agents

Given the key role of bioactive IL-1 in mediating inflammation downstream of inflammasome activation and proteolytic cleavage, blocking IL-1 signaling represents a viable therapeutic strategy [51,61,63,78]. A preclinical study demonstrated that neutralizing IL-1α reduced disease severity (IL-1α is a potent activator of fibroblast) in a mouse model of Crohn’s disease via modulation of the gut microbiome and correction of mucosal dysbiosis, suggesting potential therapeutic benefits [185]. A clinical study in very-early-onset IBD (VEO-IBD) showed positive outcomes using canakinumab, an IL-1β-neutralizing antibody [171]. No clinical trials or published studies have yet evaluated the use of canakinumab in PSC patients. While IL-1β plays a role in various inflammatory processes, its specific involvement in PSC pathology remains under-researched [186]. A study lead by Yin and colleagues showed that the immuno-blocking bispecific antibody FL-BsAb1/17, which targets IL-1β and IL-17A, reduced lesions of DSS-induced ulcerative colitis in mice and significantly reduced the degree of fibrosis in vivo, suggesting that IL-1 and/or IL-17 are pivotal contributors to inflammatory and fibrotic diseases such as PSC and IBD [164]. Nevertheless, the efficacy and safety profile of IL-17-targeting approaches remains controversial in regards of IBD as secukinumab, a human anti-IL-17A monoclonal antibody, has been reported to be a potential trigger of intestinal mucosal inflammation when being used in the treatment of other IMIDs such as in ankylosing spondylitis or psoriatic arthritis patients [187,188]. Moreover, secukinumab treatment was ineffective in a randomized, double-blinded placebo-controlled trial in moderate-to-severe CD [189].

### 8.4. IL-1R1/2 Signaling Interference

A systematic review highlighted that IL-1R-targeting biologics, including anakinra—a recombinant human IL-1R1 antagonist that antagonizes bioactive IL-1α and IL-1β molecules—have been beneficial in treating rheumatoid arthritis and other autoinflammatory conditions such as periodic fever with aphthous stomatitis, pharyngitis, and adenitis (PFAPA) syndrome, hyper-IgD syndrome (HIDS), cryopyrin-associated periodic syndromes (CAPS), and familial Mediterranean fever (FMF)—syndromes that are associated with mucosal manifestations [30,190,191]. Therefore, anakinra has been considered for its potential to modulate inflammation in PSC and forms of IBD [192]. Promisingly, anakinra-mediated IL-1R blockade reduced intestinal inflammation in a murine model of TNF-independent UC [165]. To this end, a comparative study about the efficacy of inflammasome signaling inhibitors in a preclinical murine model of colitis showed positive effects of anakinra [193]. Nonetheless, in patients with IBD, the effect of anakinra is controversial as a trial of anti-IL-1 therapy in acute fulminant UC was stopped due to futility and case report data of a patient with FMF (as a rheumatic condition) and UC showed divergent effects of IL-1R1 blockade, highlighting the potential importance of considering the disease context regarding anakinra administration as well as the urgent need for further and larger trials with various IBD severities, phenotypes, and disease states [174,194,195]. Nevertheless, IL-1R1 blockade with anakinra has shown efficacy in mucosal healing in some pediatric patients with IL-10 receptor deficiency—a monogenic form of VEO-IBD marked by enhanced macrophage-mediated IL-1 production and an aggressive, Crohn’s-like phenotype with deep ulceration [172,173]. Furthermore, research indicates that IL-1R2, a decoy receptor for IL-1, is being upregulated during remission in ulcerative colitis. This suggests that IL-1R2 may act as a homeostatic regulator, with its expression potentially linked to disease remission [196,197]. IL-1R2 may therefore represent a valid therapeutic target. While IL-1 family members have been implicated in liver diseases, specific studies focusing on therapeutic approaches targeting IL-1R1 and IL-1R2 in PSC are currently lacking [198]. Other promising approaches that have been described are the oral administration of pH-sensitive microcapsules containing an endogenous IL-1R1 antagonist (IL-1Ra) as well as genetically modified lactic acid bacteria (*Lactococcus lactis*) engineered to synthetize and secrete IL-1Ra. Both showed promising anti-inflammatory effects in an animal model of acute colitis [166,167,199]. Clinical evidence of such IL-1R1-blocking avenues in PSC is currently limited and remains to be explored.

### 8.5. Interleukin-18 Inhibitors

One preclinical study demonstrated that the administration of an IL-18 binding protein (APB-R3) effectively alleviated biliary injuries in a 3,5-Diethoxycarbonyl-1,4-Dihydrocollidine (DDC) diet-induced rodent model of PSC, suggesting IL-18 signaling as a potential therapeutic target in PSC [200]. In vivo, administration of an IL-18BP alleviated DSS-induced colitis [201] and single knockout of IL-18 (as well as double knockout together with IL-1β) protected against dinitrobenzene sulfonic acid (DNBS)-induced colitis [202]. Moreover, recent findings show that monoclonal antibodies against IL-18 can ameliorate intestinal inflammation by restoring goblet cell function and repairing the mucus layer in vivo [203]. Together, these findings underscore the relevance of IL-1 superfamily members—particularly IL-18—in PSC and IBD pathogenesis [204].

### 8.6. Interleukin-33/ST2 Signaling Blockade

IL-33, an IL-1 superfamily member, also seems to play a key role in maintaining normal intestinal homeostasis by maintaining tight junctions in the intestinal epithelial layer [205]. One preclinical study showed that blocking the IL-33/suppression of tumorigenicity 2 (ST2, also known as IL-1RL1) signaling axis enhances mucosal healing in DSS- and 2,4,6-trinitrobenzene sulphonic acid (TNBS)-induced colitis in mice [206,207,208]. The IL-33/ST2 axis is reported to correlate with disease severity in hepatobiliary inflammatory and progressive fibrotic diseases such as PBC and biliary atresia, respectively [209,210]. Although it therefore appears likely that IL-33/ST2 signaling might represent a feasible target in PSC and IBD, there is no published data that reports the effect of an IL-33/ST2 signaling blockade on fibrosis in either PSC or IBD to our knowledge whatsoever.

### 8.7. Serum Amyloid A Targeting Therapeutics

SAA, as part of an inflammatory response, is primarily released into the circulation by hepatocytes under the transcriptional regulation of IL-1 and other pro-inflammatory cytokines [112]. Several studies have shown that excessive SAA promotes inflammatory diseases by activating innate immune cells and enhances expression and activation of inflammasomes through TLR and P2X7 receptor signaling, thus acting as a DAMP in such contexts and causing an amplification of inflammatory processes [120,121,123]. Therefore, any drug targeting IL-1 signaling/inflammasome activation is expected to lower SAA levels and vice versa. In IBD, disease activity correlates positively with SAA levels [211,212]. Given its functional role and potential as biomarker, targeting SAA in IBD and PSC appears to be a reasonable therapeutic approach. Nevertheless, direct therapeutic targeting of SAA and its pro-inflammatory effects are still under investigation in both IBD and PSC and may potentially become a focus of future research.

### 8.8. Microbiome Alterations and Fecal Microbiota Transplantation

The gut microbiome and its compositional alteration play a significant role in modulating immune responses and have been implicated in the pathogenesis of both PSC and IBD [142,143]. Inflammasome activation by bacterial products promotes inflammatory cell recruitment and regulates immune responses in tissues such as the gastrointestinal tract and hepatobiliary system. Accordingly, therapies aimed at altering gut microbiota composition have been investigated as potential treatments to modulate and reduce inflammation in IMIDs. Fecal microbiota transplantation (FMT), as a potent tool to manipulate the microbiome, involves the transfer of stool from a healthy donor to the gastrointestinal tract of a patient, aiming to restore a balanced microbiome. The FARGO clinical trial (FAecal microbiota transplantation in primaRy sclerosinG chOlangitis) investigates the use of FMT specifically in patients with PSC and IBD, assessing whether gut microbiota perturbations can influence disease progression by modulating immune responses, including inflammasome activity [213]. While the clinical study is ongoing (NCT06286709), preliminary reports suggest potential benefits, though more rigorous analyses are required.

### 8.9. Probiotics

It has been described that anti-inflammatory biologics improve intestinal dysbiosis in IBD [214]. The administration of beneficial bacteria, or probiotics, has therefore been explored as a means to modulate the gut microbiome and reduce inflammation, likely by attenuating inflammasome activity. Certain probiotic strains have demonstrated anti-inflammatory effects in preclinical models of IBD by inhibiting NLRP3 inflammasome activation. In a phase I clinical trial in PSC (NCT05053165), administration of the probiotic LB-P8 (*Leuconostoc citreum* strain) showed promising results and received fast-track FDA status. It had been suggested in animal studies that other bacterial strains such as Lp2 (*Lactobacillus plantarum* strain) have shown potential to reduce liver inflammation and oxidative damage in PSC models [215]. In IBD, especially in UC, meta-analyses of randomized controlled trials suggest that bacterial strains such as *Bifidobacteria* and *Lactobacilli* are effective—particularly multi-strain formulations—to reduce symptoms, promote remission, and improve quality of life [216,217,218]. Nevertheless, in PSC-IBD, one small randomized placebo-controlled crossover study that investigated four *Lactobacillus* and two *Bifidobacillus* strains did not show any changes in pruritus, fatigue, stool frequency, or biochemical profiles (i.e., alkaline phosphatase (ALP), gamma-glutamyl transferase, liver transaminases, bile salts, or albumin) during either placebo or probiotic administration [219]. This suggests that the effect of probiotics in PSC treatment may be limited. However, more randomized controlled trials are needed in PSC.

### 8.10. Farnesoid X Receptor (FXR) and G Protein-Coupled Bile Acid Receptor 1 (TGR5) Bile Receptor Modulation

Small molecule compounds are particularly attractive for treating IBD and PSC due to their oral bioavailability, their relatively low molecular weight, and their capacity to target intracellular signaling pathways. Two such molecules, obeticholic acid (OCA) and INT-747, are agonists of the FXR, a bile acid receptor. FXR is a nuclear receptor that regulates bile acid homeostasis. FXR signaling yields anti-inflammatory effects and is therefore an intestinal innate immunity modulator [220]. OCA-mediated FXR activation has been shown to reduce inflammasome activity, liver inflammation, and fibrosis in preclinical models [169,221]. Moreover, FXR signaling mediates reduction in IL-17 production and inflammation in IBD models [222], antagonizes macrophage-dependent licensing of effector T cells in PSC, and shows promising results in treating cholestatic liver disease [149,150]. A randomized, placebo-controlled, phase II study showed a reduction in ALP in PSC patients upon OCA administration [175,176]. It is currently approved for use in PSC in certain regions, though its role as a first-line treatment remains a subject of ongoing investigation [223]. In IBD, FXR activation inhibited inflammation and preserved intestinal barrier function *in vitro* and *in vivo* models [168]. To date, though, no clinical trials have assessed OCA effectiveness in IBD patients. 

Another bile acid receptor, G protein-coupled bile acid receptor 1 (also known as GPBAR1 or TGR5), and its mediated signaling cascade have been implicated in inflammasome inhibition by PKA phosphorylation [151]. Furthermore, GPBAR1 expression was reduced in both PSC patients and murine models of PSC, potentially contributing to an inflammatory state due to a loss of GPBAR1-mediated inflammasome inhibition [224]. The natural compounds total astragalus saponins (TAS) have recently been shown to reduce fibrosis, as well as IL-1β and IL-6 expression by reduction in NF-κB p65 phosphorylation (by TGR5 upregulation) in murine PSC models [170]. Similar findings were reported in IBD patients, where altered fecal bile acid profiles correlated with *TGR5* expression [225], and in a murine DSS-induced colitis model, where genetic ablation of *Tgr5* exacerbated colitis severity [226]. These findings highlight the protective roles of bile acid receptor agonists in diminishing chronic inflammation and fibrosis—presumably by attenuating inflammasome activity—in hepatobiliary and gastrointestinal disorders such as PSC and IBD.

## 9. Future Research Directions

Although advances have been made in understanding the molecular and immunological mechanisms underlying PSC and IBD, the development of effective treatments is an ongoing endeavor. To investigate inflammasome-driven chronic inflammation and fibrosis, experimental models have been instrumental in elucidating underlying mechanisms in PSC and IBD. Murine models, such as *Mdr2/Abcb4* knockout models, mimic key features of human PSC, including bile duct inflammation and fibrosis. Bile duct ligation (BDL) and chemical-induced models (DDC-fed rodents) replicate cholestasis and oxidative stress, allowing investigation of cholangiocyte injury and inflammasome activation. To model IBD, murine models such as DSS-, DNBS-, and TNBS-induced colitis are the most prominent models using chemical compounds. T cell transfer into recombinant activating gene (*Rag*) 1^-/-^ animals or IL-10 deficiency are other murine colitis models commonly used. These models combined with germ-free mice have value in studying the gut microbiome’s role in liver and gut immune activation and inflammation. Future research might also focus on studies that use in vitro and ex vivo models such as liver and intestinal organoids or human cholangiocytes and intestinal mucosal cells—either primary or immortalized from PSC and IBD patients. Controlled environments such as co-cultures including gut-on-a-chip models with hepatic or lamina propria immune cells may provide valuable information about temporal and spatial intercellular crosstalk in stromal compartments [227,228]. Furthermore, the panoply of available experimental platforms could also be harnessed to investigate novel therapeutic strategies such as antisense oligonucleotides (ASOs), small interfering RNA (siRNA), messenger RNA (mRNA)-based approaches, aptamers, and gene-editing approaches to target ASC-dependent inflammasome pathways in future studies of IBD and PSC. 

In addition, to the existing knowledge that (myo)fibroblast-mediated ECM deposition can be induced by various cell types and cytokines—including members of the IL-1 superfamily—it is conceivable, though not yet mechanistically proven, that IL-1R1 signaling orchestrates epithelial-to-mesenchymal transition (EMT) and/or endothelial-to-mesenchymal transition (EndoMT) through the upregulation of key transcriptional regulators, thereby enabling stromal niche reprogramming and cell differentiation associated with fibrosis in PSC and IBD [99,148,229,230,231,232,233].

Although cytokine inhibitors of various kind have demonstrated efficacy in other inflammatory conditions, their application and potential synergistic effects in PSC remain under investigation. As there are no human data that specifically evaluated ASC-targeted therapies in PSC to our knowledge, future studies might reveal novel insights into disease pathology.

## 10. Conclusions and Clinical Recommendations

PSC and IBD are chronic IMIDs characterized by progressive inflammation and stromal alterations in the liver and the gastrointestinal tract, respectively. Both diseases are driven by complex interactions among genetic predisposition, microbial dysbiosis, and immune dysregulation. A growing body of evidence highlights the central role of the innate immune system and inflammasomes—particularly ASC-dependent IL-1-producing inflammasomes—in orchestrating these inflammatory responses. Therefore, targeting specific inflammatory signaling pathways—including their downstream cytokines and receptors—represents a promising therapeutic avenue for PSC and IBD across the gut–liver axis. Preclinical evidence supports the potential of ASC blocking/interfering agents, NLRP3 and caspase-1 inhibitors, IL-1/IL-18/IL-33 targeted therapies, bile acid signaling, and microbiome-modulating strategies mainly in IBD. It may well be that assessing the expression levels of inflammasome pathway genes supports available therapeutic selection. Moreover, due to the inactive “pro-forms” and dichotomous functions of IL-1 and IL-18 in inflammatory disease pathogenesis as well as tissue regeneration, it may well be that assessing such bioactive proteins from patient-derived biopsies might guide future diagnostic strategies and personalized decision-making—such as dosing and agent selection—as restoring delicate cytokine homeostasis along the gut–liver axis appears to be crucial for positive treatment results with ASC-inflammasome targeting agents. Nevertheless, in IBD, the effects of such agents vary in clinical trials and further studies and biomarker investigations are needed to stratify responders from non-responders. In PSC, more pre- and clinical studies are required to assess the safety and efficacy of such agents in disease management. At present, there is insufficient evidence to apply these agents specifically for PSC outside the context of coexisting IBD. 

As research progresses, patient-tailored approaches based on individual molecular tissue profiling may help to develop and administer more effective and precise therapies for PSC and IBD patients in near future.

## Figures and Tables

**Table 1:** Selection of recent and promising agents targeting ASC-inflammasomes and Interleukin-1 signaling pathways in IBD and PSC. The upper panel shows preclinical data and the lower panel displays clinical studies with their main read-outs.

Agent/Drug	Disease and Specimen	Main Read-Outs	Target/Mechanism	Reference(s)
**Preclinical studies**				
MCC950	IBD; Murine	Biochemical markers (i.e., cytokine assessment), disease activity score	Inhibits ASC oligomerization	[158,159]
Decursinol Angelate (DA)	IBD; Murine	Biochemical markers (i.e., cytokine, enzyme, and metabolite assessment), histology, gene expression, disease activity score	Suggests disruption of NLRP3–Caspase-1 interaction	[160]
Atranorin	IBD; Murine	Biochemical markers (i.e., cytokine, enzyme, barrier, and signaling protein assessment), colon length, intestinal barrier permeability, histology, gene expression, disease activity score	Inhibits ASC oligomerization	[161]
Compound 10 v	IBD; Murine	Biochemical markers (i.e., cytokine, inflammasome, and signaling protein assessment), colon length, fecal blood index, body weight loss, histology	Blocks NLRP3-ASC and AIM-2-ASC interaction and STAT1/5 signaling pathways	[162]
Belnacasan (VX765)	IBD; Murine	Biochemical markers (i.e., Cytokine, inflammasome and signaling protein assessment), colon length, body weight loss, histology	Caspase-1 mediated pyroptosis suppression	[163]
FL-BsAb1/17	IBD; Murine	Biochemical markers (i.e., cytokine, enzyme, apoptosis protein, metabolite assessment), colon length, histology, apoptosis and cytokine gene expression, disease activity score	Bispecific IL-1β and IL-17 neutralizing antibody	[164]
Anakinra	IBD; Murine	Biochemical markers (i.e., cytokine, chemokines, signaling protein assessment), cytokine, chemokine and signaling gene expression, cellular IFNγ T and Foxp3+ Treg cells, histology score, colon length, body weight loss, disease activity index	IL-1R1 blockade, including TNF-independent models	[165]
Microcapsules or genetically modified *Lactococcus lactis*	IBD; Murine	Biochemical markers (i.e., cytokine and enzyme assessment), cellular L-17A positive T cells assessment, body weight loss, disease activity index, histology	IL-1Ra-containing capsules or -secreting microbiota	[166,167]
INT-747	IBD; Murine, enterocyte-like cells, patient-derived lamina propria mononuclear cells, monocytes and dendritic cells	Biochemical markers (i.e., cytokine analysis), body weight loss, epithelial permeability, rectal bleeding, colon length, ulceration status, goblet cell loss, histology (i.e., immune cell infiltration)	Modulation of inflammasome activation by FXR agonism	[168]
Obeticholic acid (OCA) containing nanoparticles	PSC; Murine, patient-derived organoids	Biochemical markers (i.e., cytokine, chemokine, signaling protein and enzyme analysis), body weight, histology (i.e., fibrosis, macrophages, ROS levels, apoptosis markers), liver injury score	Modulation of inflammasome activation by FXR agonism	[169]
Total astragalus saponins (TAS)	PSC; Murine	Biochemical markers (i.e., cytokine assessment), cholestasis parameter, histology (i.e., collagen deposition, ductular rection and fibrosis)	IL-1β and IL-6 expression downregulation by TGR5 upregulation and reduced NF-κB p65 phosphorylation	[170]
**Clinical studies**				
Canakinumab	IBD; Human Phase II	Clinical activity index, biochemical markers (i.e., CRP)	IL-1β neutralization	[171]
Anakinra	IBD; Human Phase I	Clinical symptoms, histology	IL-1R1 blockade	[172,173]
	IBD; Human Phase II	Clinical symptoms, histology, endoscopy scoring		[174]
Obeticholic acid (OCA)	PSC; Human Phase II	Biochemical markers (i.e., ALP)	Modulation of inflammasome activation by FXR agonism	[175,176]
